# The Role of Active Coping in the Relationship Between Learning Burnout and Sleep Quality Among College Students in China

**DOI:** 10.3389/fpsyg.2020.00647

**Published:** 2020-04-30

**Authors:** Yi Wang, Huiwen Xiao, Xiaotian Zhang, Li Wang

**Affiliations:** ^1^Faculty of Education, University of Macau, Macau, China; ^2^School of Psychology, Fujian Normal University, Fuzhou, China; ^3^School of Psychology, Beijing Sport University, Beijing, China

**Keywords:** sleep quality, coping, learning burnout, college student, well-being

## Abstract

Learning burnout negatively influences students’ learning and well-being. Sleep quality is directly related to students’ health and learning outcomes. Research indicates that sleep quality and coping style may be associated with learning burnout. However, the interrelationship among learning burnout, sleep quality, and coping style has not yet been fully studied. This study aimed to explore the relationship between sleep quality and learning burnout and examine whether coping mediates this relationship in Chinese university students. A total of 228 undergraduate students were recruited to participate in this research. The Simplified Coping Style Questionnaire (SCSQ), Learning Burnout Questionnaire (LBQ), and Pittsburgh Sleep Quality Index-Chinese (PSQI-C) were employed to collect data. The results showed the following: (1) poor sleep quality had a positive association with learning burnout, and (2) active coping style mediated the effects of poor sleep quality on learning burnout and the dimensions of learning burnout (depression and low sense of achievement). The findings of the current study contribute to knowledge of learning burnout and provide theoretical evidence for further educational interventions.

## Introduction

University life is rewarding yet challenging, and students may experience many difficult periods and mixed emotions. Especially in regard to academic challenges, researchers have repeatedly found that learning burnout has become a common problem among university students during their educational experience (Lin and Huang, 2012; Chunming et al., 2017; Xu, 2017). Learning burnout can be regarded as an extension of burnout and refers to negative learning mindset, attitudes, and behaviors toward study due to pressure or a lack of learning motivation, which makes people tired (Schaufeli et al., 2002; Zhang et al., 2007). Similar to the concept of burnout, the meaning of learning burnout, which refers more specifically to burnout in academics, has been considered to include emotional exhaustion, cynicism, and low efficacy (Maslach and Jackson, 1981; Lin and Huang, 2014; Ling et al., 2014). Usually, learning burnout varies by gender, with women being more prone to learning burnout (Castellanos, 2019; Templeton et al., 2019). Higher learning burnout in students has been found to have stronger effects on school achievement and even lead to drop out (Fiorilli et al., 2017). Therefore, it is important to determine the potential variables relating to students’ learning burnout in universities and identify means of improving this situation.

The experience of learning burnout has been found to be pervasive among university students across cultures (Ling et al., 2014; Stoliker and Lafreniere, 2015; Vahabi et al., 2018; Zhang, 2019), with negative psychological and behavioral consequences such as depression, anxiety and stress (Koutsimani et al., 2019; Mufarrih et al., 2019); low self-concept and engagement in learning (Widlund et al., 2018); and low academic achievement (Fiorilli et al., 2017). In particular, several studies conducted with Chinese samples have indicated the alarming prevalence of learning burnout in the college student population. For example, Yan et al. (2013), who conducted a survey of universities in Nanjing, China, found that 90.3% of students felt tired of learning. A range of factors that can cause learning burnout, including perceived academic stress, loneliness, and poor sleep quality, have been identified in the literature (Gibbons, 2010; Harvey et al., 2014; Lin and Huang, 2014; Stoliker and Lafreniere, 2015). To better understand the influential factors of learning burnout, a large amount of research has also been conducted to investigate the correlates of this syndrome, such as parenting style and negative perfectionism (Ding et al., 2019), education achievement attribution and academic self-efficacy (Song and Luo, 2018), with intrinsic motivation and teacher affective support (Karimi and Fallah, 2019), and sleep quality and coping style (Gibbons, 2010; Azizollah et al., 2015). Among these correlations, the positive relationships between learning burnout, stress, and loneliness have been extensively discussed (e.g., Lin and Huang, 2012; Fares et al., 2016; Worly et al., 2019). To our knowledge, however, no study has been conducted to assess the relationship between learning burnout, coping style, and sleep quality among Chinese university students. Thus, it is worth surveying how the aforementioned factors affect learning burnout in China.

Sleep is a periodic resting condition of the body and the nervous system that is crucial for university students’ learning, performance, and health (Suen et al., 2008; Simon et al., 2020). In particular, poor sleep quality, which is a recurring feature of student life that may affect not only cognitive processes but also recovery from stress and the elimination of fatigue, has been found to be associated with many psychological factors (Lin and Huang, 2012). A previous study also showed that sleepiness increased the possibility of poor school performance (Dewald-Kaufmann et al., 2010; Lin and Huang, 2014), which may cause learning burnout. A few attempts have been made to investigate the relationship between sleep quality and learning burnout. For example, Pagnin et al. (2014) found that learning burnout and sleep disorders showed relevant bidirectional effects in medical students in the early phase of medical school (Pagnin et al., 2014). Furthermore, some studies have shown that the poorer sleep quality people have, the more they experience performance failure and learning burnout (Azizollah et al., 2015). This study explored the relationship between sleep quality and learning burnout among students in universities and the mediating mechanism in this relationship.

As the influencing factor of learning burnout and sleep quality, coping style refers to individuals’ behavioral and cognitive attempts to overcome or tolerate external and internal challenges or stressors (Skinner et al., 2003) and has been roughly divided into two categories: active coping and passive coping (Li et al., 2014). According to Carver (1997), when dealing with stressors, individuals adopting active coping strategies (1) consider ways to overcome stress and make plans for subsequent efforts, (2) accept the existence of stressful events, and (3) take full advantage of the situation by learning lessons from it or looking at the bright side of the situation. In contrast, individuals applying passive coping strategies (1) refuse to acknowledge the existence of stressful events, (2) give up on making efforts to pursue the goals set under stressful situations, (3) strengthen stressful feelings, and (4) make fun of the stressor. Nonetheless, as coping is highly situational (Carver and Scheier, 1994), empirical evidence indicated that active coping is not always adaptive for stressor. A meta-analysis by Clarke (2006) revealed that active coping is maladaptive when the stressor is not controllable based on one’s ability and available resources (e.g., parental discord, one’s best friend moving away). In most cases, therefore, people’s coping behaviors can be part of the explanation why exposure to the same stressors may cause burnout in some subjects but not others. For instance, Luo et al. (2016) conducted a survey of 1,112 middle school students and reported that when they encountered difficulties, students who set high standards for their performance and who highly valued self-esteem tended to adopt active coping strategies rather than attempt to escape; they subsequently experienced less learning burnout than those who exhibited the adverse way. Consistent findings have also been found in prior studies that active and effective coping could reduce burnout levels experienced by individuals (Kilfedder et al., 2001; Doolittle, 2007). In a similar vein, Gibbons’s (2010) investigation of the relationship between sources of stress and learning burnout among university students revealed that passive coping was a strong predictor of learning burnout. Limited work has been done to characterize coping strategies related to sleep quality. Hofstetter et al. (2005) examined the effects of sleep quality on the coping style and quality of life of patients with schizophrenia, and the results showed that poor sleep quality could impair coping in patients and sustain poor quality of life. Hoyt et al. (2009), on the other hand, tested the impact of coping style on sleep quality; their findings also revealed that frequent use of passive coping strategies led to severe sleep-related disturbance.

Previous research has found limited effects of the educational environment on students’ learning burnout. Instead, it has been suggested that sleep quality has a significant influence on university students’ learning burnout (Mazurkiewicz et al., 2012; Pagnin et al., 2014; Azizollah et al., 2015). Moreover, a large number of studies have suggested the potential impact of coping style on students’ learning burnout, demonstrating a close association between sleep quality and coping style as well as between coping style and learning burnout for students in universities. Thence, through deductive reasoning, the present study investigated the mediation effect of coping style on the relationship between sleep quality and learning burnout. The study hypothesized that (1) Sleep quality could negatively predict college students’ learning burnout. (2) Coping style mediated the relationship between sleep quality and learning burnout.

## Materials and Methods

### Participants

The study participants were 228 Chinese college students. The participants were sampled with an online self-report survey. In total, 300 responses to the online survey were received. In the primary analysis, after the incomplete questionnaires and outliers were removed, 228 responses remained, for a valid rate of 76%. Among the students, 81 were male (35.5%), and 147 were female (64.5%). Their ages ranged from 18 to 30 years old (*M* = 22.18). This study adhered to the ethical principles of human subjects and was approved by the Ethics Board of the School of Psychology, Beijing Sport University.

### Instruments

#### Simplified Coping Style Questionnaire (SCSQ)

Coping style was measured with the Simplified Coping Style Questionnaire (SCSQ) (Xie, 1998). This questionnaire is a four-point Likert self-report questionnaire that includes 20 items divided into two dimensions: active and passive coping styles. Participants are asked to evaluate their frequency of engagement in certain behaviors as “Never,” “Occasionally,” “Sometimes,” or “Often.” The active coping dimension is composed of items 1 to 12, which mainly reflect active coping strategies an individual use when encountering stress, such as “trying to see things in as good of a way as possible” and “identifying several different ways to solve problems.” The passive coping dimension consists of items 13–20, which mainly reflect passive coping strategies that an individual use when encountering stress, such as “relieving troubles through smoking and drinking” and “fantasizing that some miracle may happen to change the *status quo*.” The SCSQ was shown to be valid and reliable in the original study, and the Cronbach’s α of the subscales ranged from 0.73 to 0.80 in the present study. The SCSQ score reflects participants’ coping style preferences, with a higher score indicating a higher possibility that the participant would adopt the relevant coping style.

#### Learning Burnout Questionnaire (LBQ)

The Learning Burnout Questionnaire (LBQ) developed by Lian et al. (2005) was used to measure the learning burnout of college students. This scale employs a five-point Likert response scale with 20 items across three factors: a low sense of achievement (eight items), take example as “It’s not easy for me to master the professional knowledge only when I take the exam,” misconduct (six items), like “I only study when I have an exam,” and depression (six items), such as “I feel exhausted after studying all day.” Participants are asked to subjectively evaluate items in accordance with their experience using the following response options: 1—“Strongly disagree,” 2—“Disagree,” 3—“Neutral,” 4—“Agree,” and 5—“Strongly Agree.” All factor scores are summed to calculate the total score, and higher scores indicate higher levels of learning burnout, which is categorized as follows: low (<33), average (33–66), and high (>66). Regarding the overall internal consistency, the Cronbach’s α was 0.93, and for the individual dimensions, the Cronbach’s α values were 0.88 for depression, 0.79 for misconduct, and 0.81 for low sense of achievement, which proves that the scale has acceptable internal consistency reliability.

#### Pittsburgh Sleep Quality Index-Chinese (PSQI-C)

The Pittsburgh Sleep Quality Index (PSQI) was used to assess an individuals’ quality of sleep in the last 30 days. Liu et al. (1996) translated the original PSQI developed by Buysse et al. (1989) into the Chinese version of the PSQI, which includes the following seven components and uses a specific calculation formula: subjective sleep quality, sleep latency, sleep duration, sleep efficiency, sleep disturbance, and daytime dysfunction. Each component scale has a score of 0–3, with a higher total score indicating worse sleep quality. The component scale scores are summed to determine the total score, which is categorized as follows: good (0–5), fairly good (6–10), normal (11–15), and bad (16–21). The PSQI was validated by Buysse et al. (1989) and showed good reliability, with a Cronbach’s α of 0.83. The Chinese version of the PSQI was tested and found to be a valid clinical instrument with good reliability. The Cronbach’s α was 0.84 in the previous study (Buysse et al., 1989) and 0.75 in the current study.

### Procedure

The study was conducted via the Internet, and participation was voluntary. All the participants were informed about the general aim of the research and the anonymity of their data. After providing informed consent and demographic information, the participants completed the SCSQ and LBQ and then the PSQI-C. Two control questions assessing the seriousness of participation were included in this survey. The estimated completion time was approximately 15 min.

### Data Analyses

Data analyses were performed using SPSS 19.0 and AMOS 23.0. The statistical results are displayed as the mean, standard deviation, and percentage. Gender differences were analyzed using a t-test. Correlation and regression analyses were conducted to analyze the relationships between variables. The model fit of the structural equation models and the mediating effects of coping style were tested by using AMOS 23.0. To investigate whether active coping style mediated the relationship between learning burnout and poor sleep quality, a bootstrapping procedure was performed in which the 95% *CIs* and 2,000 bootstrapping samples were used. With this method, multiple samples are taken from a set of data to approach the true sampling distribution. If the *CIs* computed for the effects do not include 0, then the effects are significant (Hayes and Scharkow, 2013). First, we explored coping style as a mediator between learning burnout and poor sleep quality. Three mediation models were investigated. Poor sleep quality was the independent variable, and coping style (active or passive coping style) was the mediator. In the first model, the learning burnout total score was the dependent variable; in the remaining two models, depression and low sense of achievement were the dependent variables.

## Results

### Gender Differences in Learning Burnout, Coping, and Sleep Quality

Overall, a moderate level of learning burnout (*M* = 49.50, *SD* = 9.49) was found in Chinese college students. Regarding the dimensions of learning burnout, the depression score was moderate (*M* = 2.55, *SD* = 0.830), and the misconduct (*M* = 2.59, *SD* = 0.44) and low sense of achievement (*M* = 2.26, *SD* = 0.60) scores were not high. Gender differences in the low sense of achievement dimension of learning burnout were significant, *t* = 2.074, *p* < 0.05. Females (*M* = 2.15, *SD* = 0.50) presented lower achievement than males (*M* = 2.31, *SD* = 0.64). There were no significant gender differences in depression, *t* = 1.31, *p* > 0.05, and misconduct, *t* = 1.58, *p* > 0.05. Based on a comparison of the average coping style scores, college students tended to use active coping (*M* = 3.02, *SD* = 0.42) rather than passive coping (*M* = 2.45, *SD* = 0.49), *t* = 13.317, *p* < 0.001. There were no significant gender differences in coping in the current study (active coping, *t* = 0.408, *p* > 0.05; passive coping, *t* = 1.935, *p* > 0.05). Overall, sleep quality was fairly good (*M* = 6.99, *SD* = 2.92). Gender differences were not found in sleep quality, *t* = 0.935, *p* > 0.05. Table 1 shows the correlations between learning burnout, sleep quality, and coping style. Preliminary analysis of the correlations among variables indicated that there was a moderate positive correlation between learning burnout and sleep quality (*r* = 0.213, *p* < 0.01), with high learning burnout associated with a high level of poor sleep quality.

**TABLE 1:** The correlations among learning burnout, sleep quality, and coping styles.

Factors		1	2	3	4	5	6	7	8	9	10	11	12	13
1	AC	−												
2	PC	0.184**	−											
3	PQSI	–0.088	0.276**	−										
4	SSQ	–0.170	0.090	0.508**	−									
5	LS	–0.092	0.125	0.661**	0.481**	−								
6	SP	0.067	0.166*	0.486**	0.139*	0.159*	−							
7	SE	0.005	0.038	0.404**	−0.158*	–0.010	0.019	−						
8	SD	–0.083	0.206**	0.619**	0.214**	0.257**	0.354**	0.068	−					
9	DD	–0.113	0.200**	0.580**	0.353**	0.366**	0.121	–0.040	0.406**	−				
10	LB	−0.226**	0.327**	0.213**	0.302**	0.141*	0.120	−0.137*	0.214**	0.354**	−			
11	DP	−0.283**	0.227**	0.164*	0.263**	0.112	0.066	−0.140*	0.208**	0.333**	0.946**	−		
12	MC	0.538**	0.718**	0.244**	–0.015	0.092	0.226**	0.050	0.164*	0.123	0.186**	0.014	−	
13	LA	−0.465**	–0.082	0.080	0.322**	0.097	0.028	−0.140*	0.060	0.227**	0.753**	0.639**	−0.266**	−
	Skewness	–0.335	–0.105	0.854	0.396	0.264	0.824	0.017	0.748	0.081	0.058	0.190	0.087	0.360
	Kurtosis	–0.136	–0.228	1.023	0.727	–0.710	0.177	–1.442	0.953	–0.616	–0.608	–0.778	0.278	–0.322
	*M*	3.020	2.453	6.991	1.013	1.377	0.693	1.482	1.232	0.917	2.467	2.552	2.593	2.255
	*SD*	0.418	0.487	2.916	0.640	0.914	0.741	1.155	0.525	0.648	0.441	0.830	0.438	0.600

*p < 0.05, **p < 0.01. PC, passive coping; AC, active coping; LB, learning burnout; DP, depression; MC, misconduct; LA, low sense of achievement; SSQ, subjective sleep quality; LS, Latent sleep; SP, sleep persistence; SE, sleep efficiency; SD, sleep disorder; DD, daytime dis-function; PQSI, the pittsburgh sleep quality index.

In addition, there was a moderate positive relationship between learning burnout and passive coping style (*r* = 0.327, *p* < 0.01), with high learning burnout associated with high levels of passive coping style. Furthermore, the results suggested a moderate negative correlation between learning burnout and active coping style (*r* = −0.226, *p* < 0.01).

### Sleep Quality and Coping Style as Direct Predictors of Learning Burnout

One predictor, namely, poor sleep quality, was entered into linear regressions that were conducted to explain the relationships among learning burnout, coping, and the dimensions of learning burnout and coping (see Table 2). A stepwise regression model was proposed to explain the variation in learning burnout. The changes in the regression models are shown in Table 3. Coping style (x_2_ and x_3_) was entered into the regressions after poor sleep quality (x_1_). The equation of the regression models is as follows:

$$\begin{array}{c} Y_{1}=\beta_{0}+\beta_{1}\left(x_{1}\right)+\beta_{2}\left(x_{2}\right)+\beta_{3}\left(x_{3}\right)+e_{1}\\ Y_{2}=\beta_{0}+\beta_{1}\left(x_{1}\right)+\beta_{2}\left(x_{2}\right)+\beta_{3}\left(x_{3}\right)+e_{2}\\ Y_{3}=\beta_{0}+\beta_{1}\left(x_{1}\right)+\beta_{2}\left(x_{2}\right)+\beta_{3}\left(x_{3}\right)+e_{3}\\ Y_{4}=\beta_{0}+\beta_{1}\left(x_{1}\right)+\beta_{2}\left(x_{2}\right)+\beta_{3}\left(x_{3}\right)+e_{4} \end{array}$$

**TABLE 2:** The prediction of poor sleep quality on variables.

Predictor	Dependent variables											
	Active coping		Passive coping		Learning burnout		Depression		Misconduct		Low sense of achievement	
	*B*	*SE*	*B*	*SE*	*B*	*SE*	*B*	*SE*	*B*	*SE*	*B*	*SE*
Constant	37.585	0.616	19.07	0.483	14.988	0.375	17.651	0.798	15.619	0.327	11.695	0.424
PSQ	−0.170*	0.514	0.09	0.404	0.302***	0.313	0.263***	0.666	–0.015	0.273	0.322***	0.354
*R*^2^	0.029		0.008		0.091		0.069		0.000		0.104	
*Adjusted R*^2^	0.025		0.004		0.087		0.065		−0.004		0.100	
*F*	6.703*		1.856		22.727***		16.791***		0.050		26.160	

*p < 0.05, ***p < 0.001.

**TABLE 3:** The changes of models after enter coping styles into regression analysis.

Model	Y1		Y2		Y3		Y4	
	*B*	*SE*	*B*	*SE*	*B*	*SE*	*B*	*SE*
Constant	15.500	1.573	23.966	3.405	–0.898	0.841	23.431	1.747
PSQ	0.228***	0.294	0.188**	0.637	–0.002	0.157	0.254***	0.327
PC	0.353***	0.048	0.265***	0.105	0.641***	0.026	–0.028	0.054
AC	−0.252***	0.038	−0.300***	0.082	0.420***	0.020	−0.478***	0.042
*R*^2^		0.241		0.195		0.686		0.278
*Adjusted R^2^*		0.231		0.184		0.682		0.268
*F*		23.752**		18.060***		163.452***		28.767***
Δ*R*^2^		0.050		0.034		0.686		0.174
Δ*F*		14.624**		9.408**		245.099***		27.054***

**p < 0.01, ***p < 0.001. Y1, learning burnout; Y2, depression; Y3, misconduct; Y4, low sense of achievement; PSQ, poor sleep quality; PC, passive coping; AC, active coping.

Notes: *Y*_1_, learning burnout; *Y*_2_, depression; *Y*_3_, misconduct; *Y*_4_, low sense of achievement, b_0_, constant; β_1_, β_2_, β_3_, regression coefficients; x_1_, poor sleep quality; x_2_, active coping; x_3_, passive coping; e, error.

The results showed that poor sleep quality and coping style explained 24.1% of the variance in learning burnout, *F*_(3_, _224)_ = 23.75, *p* < 0.001. Poor sleep quality (β_1_ = 0.228, *t* = 3.824, *p* < 0.001), active coping style (β*_2_* = −0.252, *t* = 4.181, *p* < 0.001), and passive coping style (β*_3_* = 0.353, *t* = 5.917, *p* < 0.001) were associated with learning burnout. Poor sleep quality and coping style explained 19.5% of the variance in the depression dimension of learning burnout, *F*_(3_,_224)_ = 18.06, *p* < 0.001. Poor sleep quality (β*_1_* = 0.188, *t* = 3.067, *p* < 0.01), active coping style (β*_2_* = −0.300, *t* = 4.831, *p* < 0.001), and passive coping style (β*_3_* = 0.265, *t* = 4.316, *p* < 0.001) were associated with depression. Poor sleep quality and coping style explained 68.6% of the variance in the misconduct dimension of learning burnout, *F*_(3_,_224)_ = 163.452, *p* < 0.001. Active coping style (β*_2_* = 0.420, *t* = 10.823, *p* < 0.001) and passive coping style (β*_3_* = 0.641, *t* = 16.718, *p* < 0.001) were associated with misconduct, while poor sleep quality was not a significant predictor of misconduct (β*_1_* = −0.002, *t* = 0.039, *p* = 0.969). Poor sleep quality and coping style explained 27.8% of the variance in the low sense of achievement dimension of learning burnout, *F*_(3_,_224)_ = 28.767, *p* < 0.001. Active coping style (β*_2_* = −0.417, *t* = 7.091, *p* < 0.001) and poor sleep quality (β*_1_* = 0.254, *t* = 4.371, *p* < 0.001) were associated with low sense of achievement, and passive coping style did not significantly predict low sense of achievement (β*_3_* = −0.028, *t* = 0.478, *p* = 0.633).

### Active Coping Mediates the Relationship Between Sleep Quality and Learning Burnout

The indices of model fit were χ^2^, χ^2^/df, CFI, GFI, AGFI, NNFI, and RMSEA, which are common SEM fit indices. χ^2^ more easily reaches significance when the sample size (n) is more than 200 due to its sample size sensitivity. It is best if the SEM statistic χ^2^/df is less than 5, which indicates a good model fit. A CFI of more than 0.9 was used as an indicator of the acceptability of the model (Li, 2006). The GFI evaluates the closeness between a model and the observed covariance matrix. GFI, AGFI, and NNFI values greater than 0.9 are considered acceptable (Bentler and Bonett, 1980; Bentler, 1983). The RMSEA indicates how well the model fits the data, and a value below 0.8 indicates a fair fit (McDonald and Ho, 2002). The fit indices for models 1–3 are listed in Table 4.

**TABLE 4:** Indicates for three models.

Model	χ^2^	χ^2^/df	*CFI*	*GFI*	*AGFI*	*NNFI*	*RMSEA*
Model 1	102.732	5.707	0.837	0.907	0.814	0.814	0.144
Model 2	140.916	1.409	0.965	0.926	0.900	0.958	0.042
Model 3	45.539	1.084	0.995	0.965	0.945	0.993	0.019

**FIGURE 1:**
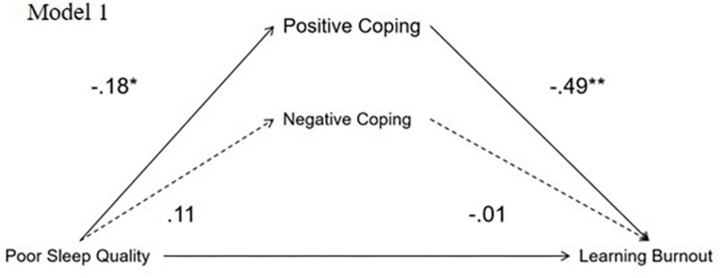
Model 1 with learning burnout as dependent variable. ^∗∗^Correlation is significant at the 0.01 level (2-tailed).

Model 1 in Figure 1, with learning burnout as the dependent variable, showed indirect effects. Poor sleep quality had a positive total effect on learning burnout (β = 0.33, *p* < 0.01, 95% CI = 0.20, 0.47). Poor sleep quality had a negative effect on active coping (β = −0.18, *p* < 0.05, 95% CI = −0.20, −0.01). Active coping had a negative effect on learning burnout (β = −0.49, *p* < 0.01, 95% CI = −8.48, −2.68). Poor sleep quality had a significant direct effect on learning burnout (β = 0.09, *p* < 0.01, 95% CI = 0.01, 0.38) and a positive indirect effect on learning burnout (β = 0.61, *p* < 0.05, 95% CI = 0.01, 0.18). Passive coping had no significant mediating effect between poor sleep and learning burnout.

**FIGURE 2:**
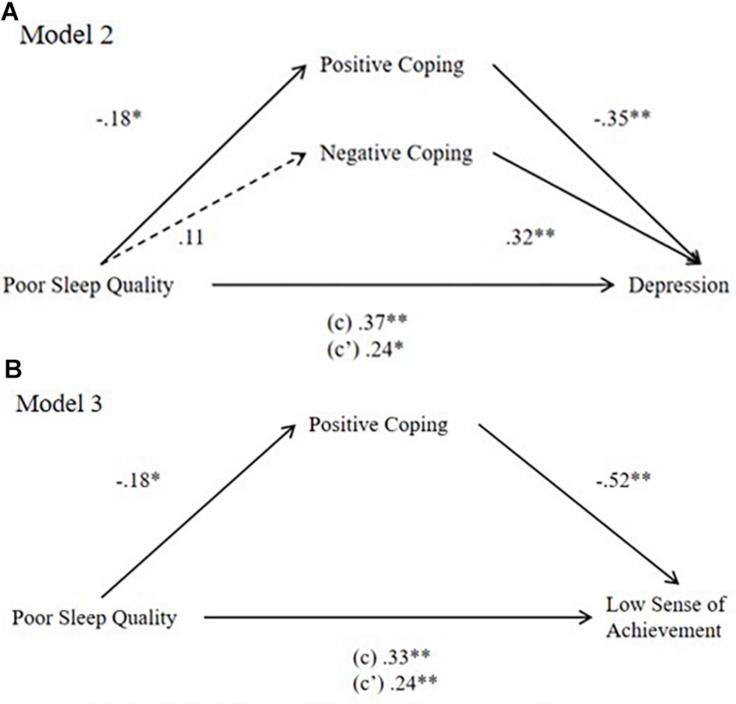
**(A,B)** Models with depression and low sense of achievement as dependent variables. * Correlation is significant at the 0.05 level (2-tailed). ** Correlation is significant at the 0.01 level (2-tailed).

Model 2 in Figure 2, with depression as an outcome, showed indirect effects. The model fit was better than that of model 1. Poor sleep quality had a positive total effect on depression (β = 0.27, *p* < 0.01, 95% CI = 0.17, 0.56) and a negative effect on active coping (β = −0.18, *p* < 0.05, 95% CI = −0.20, −0.01). Active coping had a negative effect on depression (β = −0.35, *p* < 0.01, 95% CI = −1.20, −0.48). Poor sleep quality had a significant direct effect (β = 0.24, *p* < 0.05, 95% CI = 0.04, 0.33) and a positive indirect effect (β = 0.13, *p* < 0.01, 95% CI = 0.04, 0.24) on depression.

Model 3 in Figure 2, with a low sense of achievement of learning burnout as an independent variable, showed indirect effects. The model had a good fit. Poor sleep quality had a positive total effect on low sense of achievement (β = 0.33, *p* < 0.01, 95% CI = 0.21, 0.47) and a negative effect on active coping (β = −0.18, *p* < 0.05, 95% CI = −0.20, −0.01). Active coping had a negative effect on low sense of achievement (β = −0.53, *p* < 0.01, 95% CI = −1.19, −0.56). Poor sleep quality had a significant direct effect (β = 0.24, *p* < 0.01, 95% CI = 0.11, 0.38) and a positive indirect effect (β = 0.18, *p* < 0.05, 95% CI = 0.01, 0.19) on low sense of achievement.

As mentioned before, gender differences in low sense of achievement were observed. Therefore, a multigroup invariance analysis was conducted to test the stability of model 3 to ensure that it was suitable for both males and females. The indicators shown in Table 5 illustrate that there were no gender differences in model 3.

**TABLE 5:** Gender invariance testing.

Model	χ *2*	*df*	*CFI*	*GFI*	*AGFI*	*NNFI*	*SRMR*	*RMSEA*	Δχ *2*	Δ *df*
Unconstrained	91.212	84	0.989	0.934	0.897	0.985	0.067	0.019	–	–
Fully constrained	104.105	95	0.986	0.926	0.897	0.984	0.084	0.021	12.893	11

## Discussion

In general, our study confirmed the significant relationship between burnout and negative psychological states, which has been repeatedly identified in quite a few prior studies across diverse disciplines, as reported in a recent systematic review conducted by Koutsimani et al. (2019). The previous robust evidence that paved the way for this study focused on learning burnout to explore its association with university students’ sleep quality and coping styles. This study first examined the relationship between learning burnout, sleep quality, and coping in female and male college students. The results indicated that compared to women, men scored significantly higher on low sense of achievement, one of the subscales of the LBQ. Men experienced significantly greater learning stress than women, which might have been related to their personality traits, such as high conscientiousness, while low extraversion might be associated with girls’ academic achievement (Janošević and Petrović, 2019). In this study, the hypothesis that sleep quality would negatively affect students’ learning burnout was supported. It was found that poor sleep quality was a predictor of learning burnout. Additionally, poor sleep quality was a predictor of depression and a low sense of achievement. In addition, the hypothesis that coping style would mediate the relationship between sleep quality and learning burnout was supported. Active coping style played a mediating role in the relationship between poor sleep quality and depression and in the relationship between poor sleep quality and low sense of achievement among students. Passive coping style did not have significant mediating effects on the relationship between poor sleep quality and learning burnout.

The findings from the current study echo previous studies on the topic of learning burnout. We found a weak positive correlation between poor sleep quality and learning burnout in Chinese college students. Similar findings of negative correlations between learning burnout and sleep quality were described in a previous study (Yan et al., 2018). Yan et al. (2018) found that adolescent academic stress was indirectly associated with sleep quality through the mediating effect of school burnout and depression. Previous studies have suggested that sleepiness increases poor academic performance (Pagnin et al., 2014), which leads to psychological distress and negative emotions, possibly causing learning burnout (Dewald-Kaufmann et al., 2010; Lin and Huang, 2014; Wolf and Rosenstock, 2017). However, it was found in the present study that there was no significant association between poor sleep quality and misconduct. Beebe (2011) observed that inadequate sleep could cause problems with cognition, behavior, or other functions in Western children (Beebe, 2011). Willis and Gregory (2015) also reported that poor subjective sleep quality was associated with children’s behavioral problems (Willis and Gregory, 2015). Participants in the present study were college students, who are not as sensitive as children (Park et al., 2001), and their average sleep quality was good and was not characterized as pathological. Furthermore, although misconduct is generally considered a part of burnout, research has shown that misconduct is not applicable to all situations or to all individuals (Garden, 1987). This study found that poor sleep quality could predict learning burnout, depression, and low sense of achievement but could not predict misconduct. Garden (1987) also observed that misconduct is a type-specific concept that is not applicable to the prediction of learning burnout based on sleep quality.

This study found that an active coping style may help students mediate the effects of poor sleep quality on learning burnout. Models 1–3 were consistent with the hypothesis that coping style mediates the effects of sleep quality on depression and a low sense of achievement in college students. This finding is similar to those of previous studies that revealed that sleep quality and coping style were both independent predictors of learning burnout (Pagnin et al., 2014; Vizoso et al., 2019). Sleep quality, as an important factor in students’ health, has a crucial impact on mental well-being and emotions. Poor sleep quality directly affects learning burnout and its two dimensions. A possible interpretation is that an unstable sleep schedule and sleep deprivation can lead to serious psychological and health consequences, while an optimized sleep mode can improve students’ neurocognition and academic performance (Curcio et al., 2006; Dewald-Kaufmann et al., 2010; Lin and Huang, 2014). Moreover, sleep indirectly affects learning burnout through coping: sleep influences coping style, and coping affects academic stress and improves learning burnout. Hofstetter et al. (2005) reported that poor quality sleep was negatively correlated with active coping. This study further confirmed the causal relationship between the two variables, which may be due to poor sleep quality, weakening one’s ability to find positive ways to handle stress and desire to achieve personal growth. Active coping can alleviate academic pressure and the negative effects of learning burnout among college students (Azizollah et al., 2015). Therefore, sleep can indirectly affect learning burnout through coping. Therefore, our study revealed that an active coping style played a mediating role between sleep quality and learning burnout and its dimensions, while a passive coping style did not. As in previous studies, passive coping significantly predicted depression (Guerrero, 2003; Ramírez et al., 2018; García-Arroyo and Segovia, 2019). However, no significant relationship between passive coping and sleep quality, or even a mediating effect of passive coping, was found. A previous study reported that people with poor sleep quality had more difficulty regulating their negative emotions and that passive coping could alleviate sleep disorders (Sandru and Voinescu, 2014). Unlike previous studies, this study showed that students tended to use active coping. According to Andreotti et al. (2013), active coping allows students to engage in consistent reassessments, thus, reducing the occurrence of negative results.

This study primarily examined the mediating role of active coping style between sleep quality and learning burnout and the dimensions of learning burnout in Chinese college students. The findings from this study contribute to knowledge about learning burnout and how to improve and prevent it among university populations. Foremost, the findings extend previous educational research on the association between poor sleep quality and learning burnout, which showed that these factors not only were related but also negatively affected students’ academic performance (Curcio et al., 2006). Moreover, previous scholars have focused on the relationship between the two variables, and a few have focused on the mediating effects of factors such as coping style. The present study found that coping style is a potential mediating factor of learning burnout, particularly in the relationship between sleep quality and learning burnout. Accordingly, an active coping style may be suggested as a target factor for interventions related to the influence of poor sleep quality on students’ learning burnout.

However, the present study had some limitations. One limitation was that we do not know whether the findings might have been affected by other confounding factors, such as effort control, self-efficacy, and social support (Dahlin et al., 2007; Johnson et al., 2008; Diaz et al., 2016). In addition, although the sample size of 228 was sufficient for the path analyses (which required a sample size of more than 200), it may have been a limitation within this study. Finally, the data were obtained from self-assessments that showed inaccuracies due to memory bias. As learning burnout constantly changes during a semester, a longitudinal tracking study may be needed for further exploration. Further research should be done on more than one factor. Preferably, social support should be considered a confounding factor in the relationship between learning burnout and sleep quality that may influence the levels of learning burnout. Moreover, future studies should recruit a larger sample of participants to measure group invariance by gender. Likewise, larger samples include more diversity, which could increase the generalizability of the results. Finally, future research should focus on the development and evaluation of interventions and prevention programs to accurately manage college students’ coping styles, which could reduce learning burnout.

## Conclusion

The present study demonstrated that sleep quality has a negative effect on learning burnout and the dimensions of learning burnout in Chinese college students. An active coping style mediates the effects of sleep quality on learning burnout. Therefore, promoting an active coping style and high sleep quality may effectively contribute to the improvement of learning burnout.

## Data Availability Statement

Requests to access the datasets should be directed to the first author YW (mb84822@um.edu.mo).

## Ethics Statement

This study was consistent with the ethical principles of human subjects and had been approved by The Ethics Board of the School of Psychology, Beijing Sport University.

## Author Contributions

YW, HX, XZ, and LW contributed to the conception and structure of the manuscript and wrote the manuscript. YW carried out the data collection and conducted the data analysis.

## Conflict of Interest

The authors declare that the research was conducted in the absence of any commercial or financial relationships that could be construed as a potential conflict of interest.
